# Efficient extraction of xylan from delignified corn stover using dimethyl sulfoxide

**DOI:** 10.1007/s13205-013-0159-8

**Published:** 2013-09-13

**Authors:** John Rowley, Stephen R. Decker, William Michener, Stuart Black

**Affiliations:** 1 University of Colorado, Boulder, CO USA; 2 National Renewable Energy Laboratory, Golden, CO USA

**Keywords:** Xylan, Hemicellulose, Plant cell wall, Biomass, Acetylation

## Abstract

Xylan can be extracted from biomass using either alkali (KOH or NaOH) or dimethyl sulfoxide (DMSO); however, DMSO extraction is the only method that produces a water-soluble xylan. In this study, DMSO extraction of corn stover was studied at different temperatures with the objective of finding a faster, more efficient extraction method. The temperature and time of extraction were compared followed by a basic structural analysis to ensure that no significant structural changes occurred under different temperatures. The resulting data showed that heating to 70 °C during extraction can give a yield comparable to room temperature extraction while reducing the extraction time by ~90 %. This method of heating was shown to be the most efficient method currently available and was shown to retain the important structural characteristics of xylan extracted with DMSO at room temperature.

## Introduction

Biofuels are becoming more widespread throughout the United States as more advanced conversion methods become available. The most advanced process currently is the conversion of lignocellulosic biomass into ethanol (Kim et al. 2009). Despite having much larger production potential than starch-based ethanol, lignocellulosic ethanol is still in the early stages. The conversion of biomass sugars into biofuels is an important aspect of the Department of Energy’s mission to promote the integration of renewable fuels and is a key component in the worldwide move towards renewable energy. Before additional progress can be made, it is desirable to understand in detail the mechanisms that occur during the biomass to biofuel conversion process.

Biomass is made up of three components: cellulose, hemicellulose and lignin. Xylan, a prevalent plant cell wall polymer made up of mostly xylose, is of particular interest as the dominant plant cell wall hemicellulose (Ebringerová et al. 2005). One of the challenges associated with the efficient production of biofuels involves the selective removal and/or hydrolysis the polymeric xylose backbone of xylan. During neutral or acidic thermochemical pretreatment of biomass, xylan is removed from the biomass and broken down into xylose, arabinose, and a few other minor components such as acetic acid (Naran et al. 2009).

To better understand the mechanism of thermochemical and enzymatic removal of xylan, it is useful to develop antibodies capable of tagging xylan in biomass. Antibodies can be tagged with fluorescent dyes, allowing the location of the xylan in biomass to be tracked either optically or spectrophotometrically prior to and following pretreatment. By identifying the location of the xylan, the pretreatment process and the subsequent fermentation process can be tailored to improve ethanol production. Antibody tagging can be very beneficial in understanding the mechanism of xylan removal, however, to create specific antibody tags, a native-like xylan is desirable. Many extraction methods result in degradation or de-acetylation of the xylan resulting in a non-native, water-insoluble product, which could potentially produce antibodies with non-useful specificity, as specific side groups are missing. Dimethyl sulfoxide (DMSO) extractions have been found to result in a water-soluble form of xylan, which retains the acetyl groups present in the native state (Hägglund et al. 1956). This native-like xylan is more likely to result in production of antibodies specific to the native structures found in xylans in situ in the cell wall.

In this study, a DMSO extraction of xylan in corn stover was studied at varying temperatures of extraction to determine an ideal temperature for efficient extraction.

When extracting xylan from biomass with DMSO, a pretreatment of the sample is necessary to open the cell structure and allow the polymeric xylans freedom to be extracted. Owing to the coupling between xylan and lignin, xylan is intractable until much of the lignin has been removed or these connections severed. Decoupling of xylan from lignin is important in accessing xylan in biomass, but complete removal of lignin will result in loss of xylan from the sample (Ebringerová et al. 2005). Multiple delignification procedures exist for the removal of lignin from corn stover, however, acid-chlorite bleaching was found to be the most efficient method of delignification without excessive de-acetylation of the xylan (Ebringerová et al. 2005).

Following delignification, xylan is extracted from the sample. Often xylan is extracted with KOH or NaOH (Ebringerova and Heinze 2000). However, this method results in de-esterification of the acetyl groups present on the xylan (via saponification of the ester links), leading to a water insoluble product which has limited utility for antibody production and as a substrate for hemicellulase assays. Therefore, in this study, xylan was removed by DMSO extraction to retain the acetyl groups, resulting in a water-soluble product. The extraction was first performed at room temperature, following the method proposed by Hägglund et al. (1956) in 1956. This method is carried out by stirring the biomass in DMSO for approximately 24 h at room temperature. A series of extractions was then performed at higher temperatures (70 °C and at 40 °C) with variable times of extraction. The yields resulting from the extractions were compared and, including the time required to perform each extraction, the most efficient method of extraction was determined.

Further analysis was performed on each sample to determine the content of the yield acquired through extraction and to ensure that no significant structural changes took place under heated conditions. Infrared spectroscopy and QToF MS analysis was used to determine the general structural features and to ensure that no de-esterification or de-polymerization took place during the heated extractions.

## Methods

### Delignification of biomass

Approximately 300 g of milled corn stover was extracted in a polypropylene thimble using a Soxhlet extractor following NREL’s Determination of Biomass Extractives Laboratory Analytical Procedure (Sluiter et al. 2008). The NREL procedure is a two-step procedure carried out in a Soxhlet extractor. All extractions are carried out at the reflux temperature of the solvent used and at ambient pressure. Each extraction is performed until little to no color is present in the extraction chamber. Depending on the nature of the material, this takes between 18 and 48 h for each step. The first extraction was performed with de-ionized (DI) water to remove accessible water-soluble compounds. A second extraction was performed using ethanol to remove lipids and other extractables. The solid sample was air-dried following ethanol extraction prior to delignification.

Delignification was carried out in double bagged one gallon plastic zipper closure bags by adding water to the approximately 100 g of air-dried, extracted biomass at a biomass/water consistency of 10 %. Approximately, 40 g of sodium chlorite (NaClO_2_) was added to the mixture and the bag was mixed well followed by a 5 mL addition of concentrated hydrochloric or glacial acetic acid. A smaller volume of hydrochloric acid is needed to sustain the reaction. The bag was closed and heated in a 60 °C water bath in a fume hood for approximately 3 h. Regular venting of the bag was required to relieve pressure in the bag and prevent reaching too high a concentration of ClO_2_. If the concentration of chlorine dioxide in the atmosphere of the bag or any bleaching vessel is too high, a “puff” can result from the decomposition of the chlorine dioxide. A “puff” is a term coined within the pulping industry to differentiate a low speed detonation wave of <1 m/s from an explosion wave (>300 m/s). Plastic zipper bags will open in the event of a puff releasing the gas without creating a debris hazard (Fredette 1996).

Once every hour, an additional 40 g of NaClO_2_ was added to the bag until the total amount was approximately 0.70 g NaClO_2_/g biomass. The remaining liquid was filtered from the solids and the solid biomass was thoroughly washed with DI water and lyophilized prior to DMSO extraction.

### DMSO extraction

A 1 L electrically heated reaction flask fitted with an overhead mechanical stirrer was used for all extractions. Approximately 50 g of delignified corn stover was added to a flask and extracted with DMSO using a ratio of approximately 14 mL/g biomass at room temperature with stirring at 20 rpm for a specified time. The solid was filtered and extracted a second time with DMSO for the same time period. The solid was filtered and washed thoroughly with ethanol to remove residual DMSO and extracted xylan. The ethanol filtrate was reserved for the precipitation step. The DMSO extracts were combined and absolute ethanol was added to the DMSO extract (3.8 L ethanol/L of final extract). Concentrated hydrochloric acid (HCl) was added in a ratio of approximately 0.66 mL HCl/L of ethanol/DMSO solution to precipitate the xylan from the DMSO/ethanol mixture. The solution was cooled at 4 °C overnight to complete precipitation. The cold solution was filtered though paper filter (Whatman Grade 1). The filter paper and isolated xylan were macerated, washed with ethanol and stirred overnight in a small amount of ethanol. Ethanol was filtered from the solid xylan and macerated paper filter. The resulting filter cake was stirred overnight with fresh ethanol to remove as much DMSO as possible and filtered. The filter cake was further washed with diethyl ether with overnight stirring to remove any remaining ethanol and DMSO. The xylan was dissolved away from the macerated paper fibers in warm water (30 °C), filtered with small amounts of water added for washing and lyophilized.

The DMSO extraction was carried out at 20, 40 and 70 °C according to the conditions shown in Table 1. Extractions at 40 and 70 °C were performed in duplicate. Extraction at 20 °C was a single extraction.

**Table 1:** The conditions for four subsequent extractions at temperatures above room temperature

Temperature (°C)	Time (h)	Number of extractions
70	2	2
70	2	1
70	1	2
40	4	2
20	24	2

The time noted above represents the duration of each extraction

### Sample analysis

The final products were analyzed qualitatively by their water solubility and for yield from the bleached material by mass. The methods were compared according to yield and time efficiency. Samples were analyzed on a Thermo Scientific Nicolet 6700 FTIR Spectrometer fitted with a Smart iTR diamond cell and a DTGS detector. Samples were scanned for 150 scans and compared to previously isolated and analyzed samples (Ebringerová et al. 2005).

Two samples, one extracted at room temperature, and the other at 70 °C, were prepared in a 50/50 solution of H_2_O/acetonitrile in 0.2 % formic acid. Each sample was directly infused into a Micromass Q-ToF micro (Micromass, Manchester, UK) quadrupole time of flight mass spectrometer with a 250 μL Hamilton gastight syringe (Hamilton, Reno, NV, USA) at a flow rate of 5 μL/min. Spectra were obtained in positive MS mode from a mass range of 600–1,500 *m/z* and processed by Masslynx data system software (Micromass, Manchester, OK). In positive-ion MS mode, cone voltage was set at 30 volts and capillary at 3,000 volts. Both cone and desolvation gas flows were optimized at 10 and 550 L/h, respectively. Source and desolvation temperatures were set at 100 and 250 °C. Sample mass spectra were collected for 2 min to ensure adequate signal levels. Mass calibration was performed using a solution of 2 pmol/μL of sodium rubidium iodide solution. The calibration mix was collected for 2 min and summed.

## Results

**Fig. 1:**
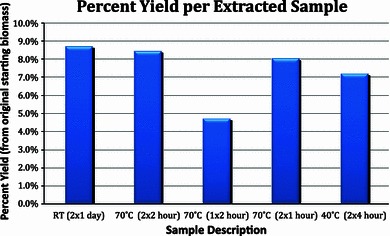
% yield of xylan extracted from bleached material. Descriptors of each sample are temperature (# replicates × time of extraction for each replicate); *RT* room temperature

Figure 1 shows the percent yield for each of the extracted samples. Analysis of the yield for the different methods indicates that heating during the extraction process results in no significant loss of recovery. It is also evident that much less time is needed for extraction when the DMSO is heated during extraction compared to a room temperature extraction. At room temperature, two sequential extractions, each lasting a full 24 h, are necessary and resulted in a xylan/delignified biomass yield of 8.7 %. Upon heating to 70 °C and decreasing the extraction time to only 2 h, the yield was 8.6 ± 0.2 %. Even when the extraction time was decreased to 1 h for a sample heated to 70 °C, the loss in yield was not found to be particularly large (7.6 ± 0.6 %). However, a significant loss in yield was found when the sample was extracted only once with DMSO. The percent yield did not drop significantly when the sample was heated to 40 °C, but a longer extraction time was necessary (Fig. 1).

**Fig. 2:**
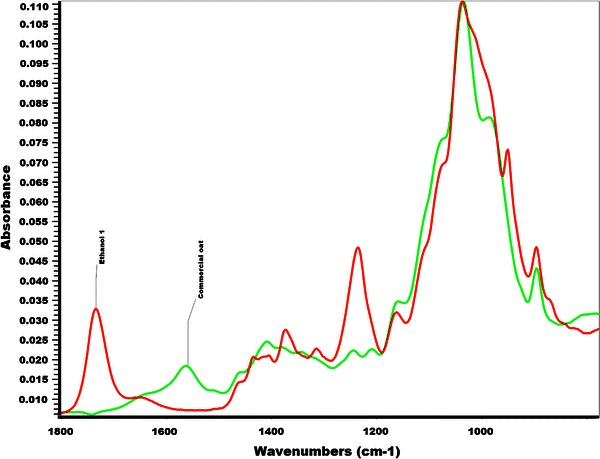
IR-spectra of xylan samples. The *red spectrum* is of the DMSO extracted xylan. The *green spectrum* is of a commercial oat xylan sample extracted with alkaline conditions. The peaks at 1,735 and 1,235/cm are indicative of carbonyl and ester linkage, respectively, of the acetyl groups on the xylan polymer

By infrared spectroscopy, there is little structural difference between the heated and room temperature extractions. Figure 2 compares corn stover xylan extracted using DMSO and a commercial oat spelt xylan (Fluka) extracted under alkaline conditions. The commercial xylan has no signal for the acetate ester present in the DMSO xylans at ~1,700 and ~1,300/cm which are the well-known carbonyl and ester linkage absorbance bands for the acetyl groups. The commercial xylan shows a slight absorbance at 1,500/cm which is indicative of residual lignin. The DMSO extracted lignin does not have an absorbance in this region. This would indicate that no either no residual lignin was present following acid chlorite delignification or that no water soluble lignin was present in the isolated xylan following lyophilization.

**Fig. 3:**
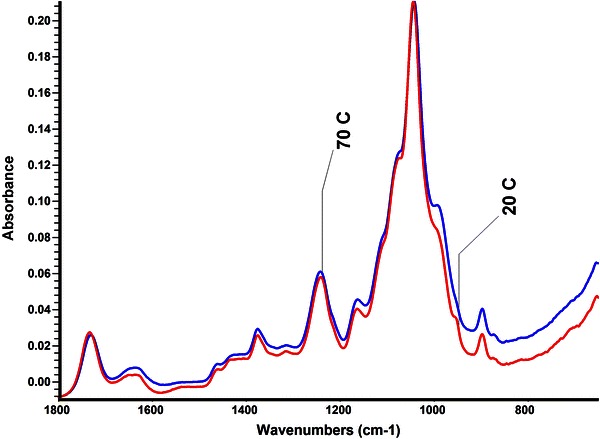
IR-spectra of two extracted xylan samples. The *blue line* is the spectrum of the DMSO extracted sample extracted at 70 °C. The *red line* is the spectrum for a corn stover extracted xylan at room temperature

When comparing (Fig. 3) the two DMSO extracted corn stover xylans, it is clear that heating during the DMSO extracting process does not influence the structure in a significant way. The expected peaks are present for the DMSO extracted sample indicating the presence of an ester group.

**Fig. 4:**
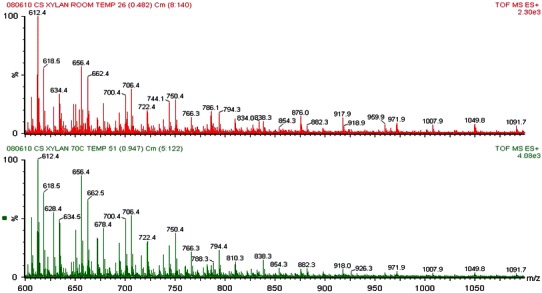
Combined mass spectra for xylan extracted at room temperature (*top*) and 70 °C (*bottom*). The combination of the spectra shows that the two samples are structurally the same

Both the room temperature- and 70 °C-extracted samples were water soluble, providing further evidence of the presence of acetyl groups on the isolated xylans, as acetylation is known to provide for water solubilization of xylans (Grondahl and Gatenholm 2005; Gabrielii et al. 2000). Figure 4 shows the mass spectra comparison between xylan extracted at room temperature and xylan extracted at 70 °C. The MS spectra collected from each sample shows a degree of polymerization range of 4–9 residues, indicative of the limitations of the ESI technique, rather than the actual DP of the sample materials. Low MW xylo-oligomer standards (DP 2–4) showed no fragmentation at the voltages used in this study (data not shown), indicating that the ions detected in the samples are generated during the sample preparation and remain with the soluble fraction during purification, not as artifacts of MS fragmentation. There are slight differences in the two spectra, specifically with intensities seen at varying masses with the relative abundance. The spectra show that a high number of the masses associated with each sample are present in the other. It is clear from the fragments in the spectra that the two xylan samples are structurally very similar, supporting the analysis from the IR instrument, and showing that no significant structural changes occurred when xylan was extracted at a higher temperature. Elucidation of the structure of the isolated xylans may be found in our previous work (Naran et al. 2009) and was not attempted in this study, which is primarily aimed at developing a faster, easier method for obtaining native xylans.

## Discussion

Drawing from these results, it can be concluded that heating during an extraction can increase the efficiency of a xylan extraction. A heated extraction requires much less time than an extraction done at room temperature. It also must be mentioned that while further study is needed, these preliminary results predict that the number of extractions (proportional to the total volume of DMSO used) does impact that percent yield. The conclusion that percent yield is increased upon heating is not supported by this study, however, further analysis can be done to confirm this prediction. These results strongly indicate that the yield obtained under heated conditions is comparable to that of an unheated extraction and requires significantly less time to extract (~9 % of the total time required to extract an unheated sample). Further, it must also be said that heating during the extraction process within the temperature range studied here does not change the xylan structure. The product is not de-esterified during the process and remains water soluble. Furthermore, no significant or obvious structural changes were observed when comparing heated samples to non-heated samples (Figs. 3, 4).

The efficiency of xylan extraction with DMSO can be greatly improved if the samples are heated during extraction. The percent yield resulting from an extraction performed at 70 °C for 2 h with multiple extractions is comparable to the yield at room temperature with two 24 h extractions. Provided that no de-esterification of the xylan results from heating the sample as shown in Fig. 3, heating increases the efficiency of the extraction. From this study, it was determined that the most efficient method of extraction is the following: two 70 °C extractions each lasting 2 h. This method provided a yield of 8.6 ± 0.2 % which is considered to be sufficient for the purposes of this study.

## References

[CR1] Ebringerova A, Heinze T (2000). Xylan and xylan derivatives—biopolymers with valuable properties, 1—naturally occurring xylans structures, procedures and properties. Macromol Rapid Comm.

[CR2] Ebringerová A, Hromadkova Z, Heinze T (2005). Hemicellulose. Adv Polym Sci.

[CR3] Fredette MC, Reeve DW, Dence CW (1996). Bleaching chemicals: chlorine dioxide. Pulp bleaching: principles and practice.

[CR4] Gabrielii I, Gatenholm P, Glasser WG, Jain RK, Kenne L (2000). Separation, characterization and hydrogel-formation of hemicellulose from aspen wood. Carbohydr Polym.

[CR5] Grondahl M, Gatenholm P, Dumitriu S (2005). Role of acetyl substitution in hardwood xylan. Polysaccharides: structural diversity and functional versatility.

[CR6] Hägglund E, Lindberg B, McPherson J (1956). Dimethylsulphoxide, a solvent for hemicelluloses. Acta Chem Scand.

[CR7] Kim TH, Nghiem NP, Hicks KB (2009). Pretreatment and fractionation of corn stover by soaking in ethanol and aqueous ammonia. Appl Biochem Biotechnol.

[CR8] Naran R, Black S, Decker SR, Azadi P (2009). Extraction and characterization of native heteroxylans from delignified corn stover and aspen. Cellulose.

[CR9] Sluiter A, Ruiz R, Scarlata C, Sluiter J, Templeton D (2008). Determination of extractives in biomass.

